# Successful repeat carotid artery replacement using vascular grafts in a case with recurrent dedifferentiated liposarcoma: a case report

**DOI:** 10.1093/jscr/rjag837

**Published:** 2026-09-22

**Authors:** Ayano Tamura, Ryuhei Okada, Ryo Wakejima, Susumu Kirimura, Kazuchika Ohno, Toshifumi Kudo, Kentaro Tanaka, Kenichi Okubo, Takahiro Asakage

**Affiliations:** Department of Otorhinolaryngology, Institute of Science Tokyo, 1-5-45, Yushima, Bunkyo, Tokyo 113-8519, Japan; Department of Head and Neck Surgery, Institute of Science Tokyo, 1-5-45, Yushima, Bunkyo, Tokyo 113-8519, Japan; Department of Thoracic Surgery, Institute of Science Tokyo, 1-5-45, Yushima, Bunkyo, Tokyo 113-8519, Japan; Department of Pathology, Institute of Science Tokyo, 1-5-45, Yushima, Bunkyo, Tokyo 113-8519, Japan; Department of Head and Neck Surgery, Institute of Science Tokyo, 1-5-45, Yushima, Bunkyo, Tokyo 113-8519, Japan; Division of Vascular Surgery, Institute of Science Tokyo, 1-5-45, Yushima, Bunkyo, Tokyo 113-8519, Japan; Reconstructive Plastic Surgery, Institute of Science Tokyo, 1-5-45, Yushima, Bunkyo, Tokyo 113-8519, Japan; Department of Thoracic Surgery, Institute of Science Tokyo, 1-5-45, Yushima, Bunkyo, Tokyo 113-8519, Japan; Department of Head and Neck Surgery, Institute of Science Tokyo, 1-5-45, Yushima, Bunkyo, Tokyo 113-8519, Japan

**Keywords:** dedifferentiated liposarcoma, local recurrence, vascular graft, vascular reconstruction

## Abstract

Dedifferentiated liposarcoma is prone to local recurrence, and complete resection remains the treatment of choice. We report a 70-year-old man who underwent carotid artery replacement with vascular grafts on two occasions for recurrent disease. A recurrent tumor invading the right common carotid and subclavian arteries was resected with vascular graft replacement. After 2 years and 7 months, recurrence developed around the carotid artery anastomosis. En bloc tumor resection with repeat carotid artery replacement using a prosthetic vascular graft was successfully performed. The patient was discharged on postoperative Day 9 without postoperative neurological deficits. Postoperative radiotherapy was administered following both operations. He remains free of recurrence more than 2 years after the most recent surgery. This case suggests that repeat carotid artery replacement using a vascular graft may be a feasible treatment option for selected patients with recurrent dedifferentiated liposarcoma.

## Introduction

Mediastinal liposarcomas are relatively rare, accounting for <1% of all liposarcomas and 0.1%–0.75% of all mediastinal tumors [1, 2]. Liposarcomas are classified into 5 types: well-differentiated liposarcoma, dedifferentiated liposarcoma, myxoid liposarcoma, pleomorphic liposarcoma, and myxoid pleomorphic liposarcoma. Dedifferentiated liposarcomas are high-grade and aggressive malignancies [3]. The therapeutic modality of choice for liposarcomas is complete resection [1, 2, 4, 5]. Neither chemotherapy nor radiotherapy has been shown to be effective, although there are several reports suggesting that adjuvant radiation therapy can improve the prognosis [4, 6]. While few studies have examined the long-term prognosis of mediastinal dedifferentiated liposarcomas, there are several reports of recurrence of these tumors [4, 6–8]. Mediastinal liposarcomas sometimes elongate surrounding aortas and veins, so that tumor resection along with the vessels and vascular replacement may need to be considered [2, 9, 10]. Herein, we report a case of recurrent liposarcoma that developed around the carotid artery anastomosis after tumor resection with carotid artery reconstruction using a vascular graft, that was successfully treated by repeat resection and repeat carotid artery replacement with a vascular graft.

## Case presentation

**Figure 1:**
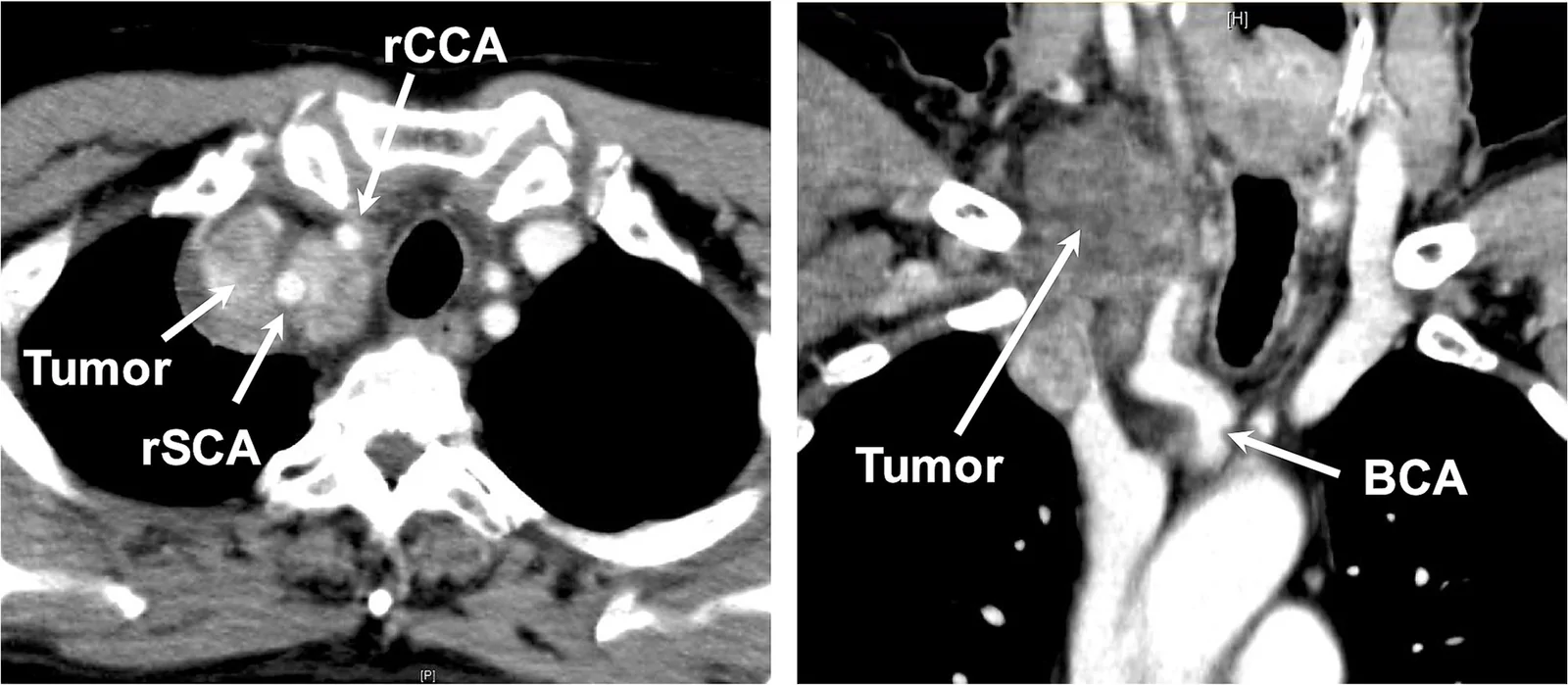
Contrast-enhanced CT before our first operation. The tumor surrounded the right subclavian artery and right common carotid artery. BCA, brachiocephalic artery; rCCA, right common carotid artery; rSCA, right subclavian artery.

A 70-year-old man who had undergone resection of a mediastinal dedifferentiated liposarcoma 10 years ago at another hospital was referred to our hospital because of recurrence of the tumor. Contrast-enhanced computed tomography (CT) revealed a giant tumor measuring 86 × 58 × 44 mm in size in the neck and superior mediastinum (Fig. 1). The tumor surrounded the right subclavian artery and right common carotid artery. The preoperative Balloon Matas Test revealed no development of neurological symptoms when the affected arteries were occluded.

**Figure 2:**
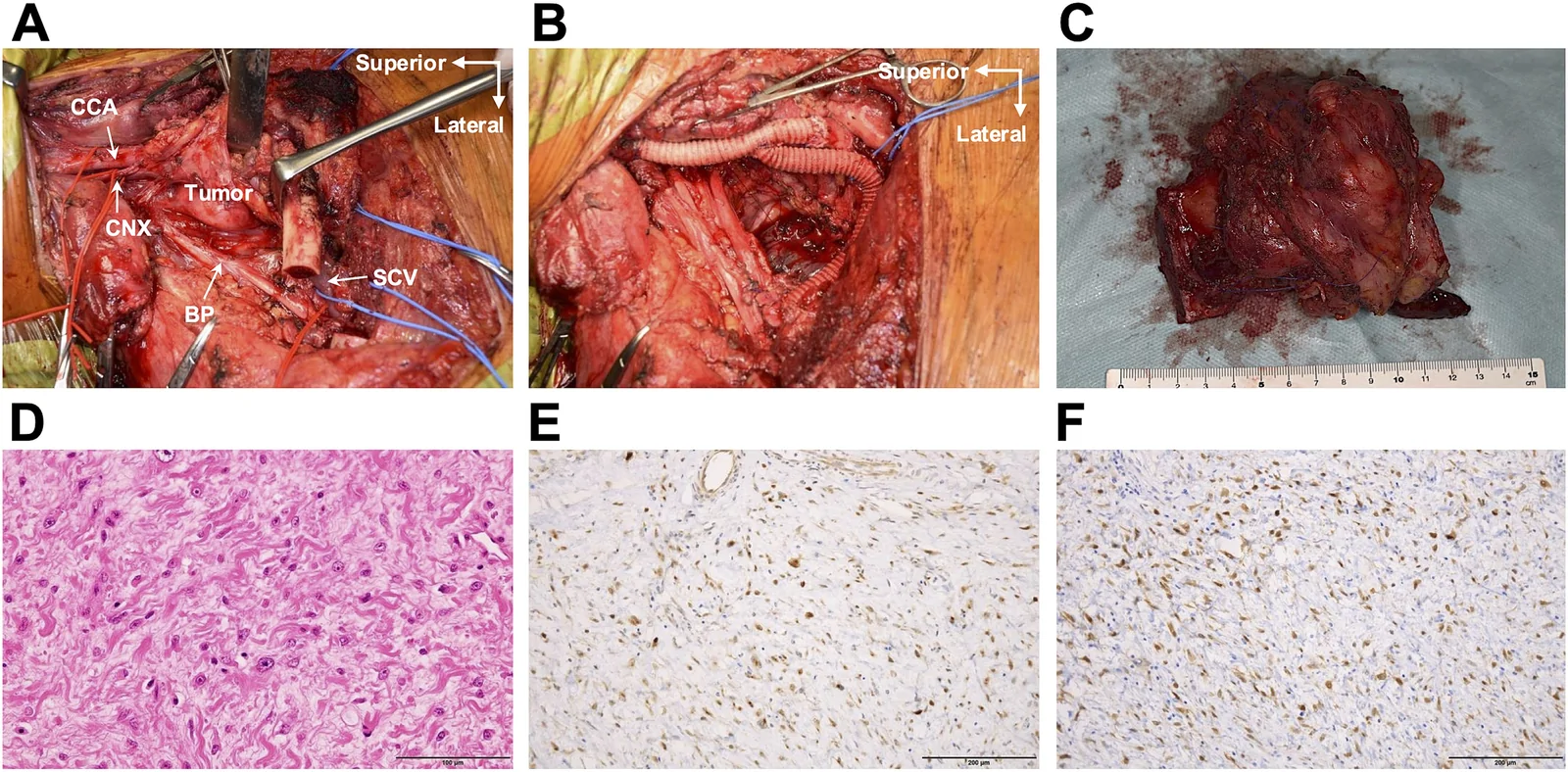
Surgical and histopathological findings at our first operation. (A) Before resection. The tumor invaded the arteries. (B) After tumor resection and vascular replacement. The arteries were reconstructed in a T-shaped configuration using vascular grafts. (C) Tumor image. The tumor measured 110 mm in maximum diameter. (D) Hematoxylin and eosin staining. Spindle-shaped tumor cells were observed. (E, F) Immunohistochemical analysis showed positive staining for MDM2 (E) and CDK4 (F). BP, brachial plexus; CCA, common carotid artery; CNX, vagus nerve; SCV, subclavian vein.

We performed tumor resection with the cervical and thoracic approach (Fig. 2A). The right vagus nerve and right phrenic nerve were also resected. Furthermore, the brachiocephalic artery, right subclavian artery and right common carotid artery were also resected and replaced with a woven gelatin-coated vascular prosthesis (J-graft, 9-mm, Japan Lifeline) in a T-shape (Fig. 2B). The prosthetic graft was covered with a pectoralis major muscle flap and the wound was closed. The tumor was 90 × 60 × 110 mm in size (Fig. 2C). Postoperative histopathology revealed spindle-shaped tumor cells (Fig. 2D). Immunohistochemistry revealed positive staining of the tumor cells for MDM2 (Fig. 2E) and CDK4 (Fig. 2F), and the tumor was diagnosed as a recurrent dedifferentiated liposarcoma. After surgery, the patient had no neurological symptoms, except for right recurrent laryngeal nerve palsy. He was discharged on postoperative Day 35. Because the postoperative histopathological examination revealed positive surgical margins at multiple sites, we administered postoperative radiotherapy to the upper mediastinum and right supraclavicular region (60 Gy in 30 fractions).

**Figure 3:**
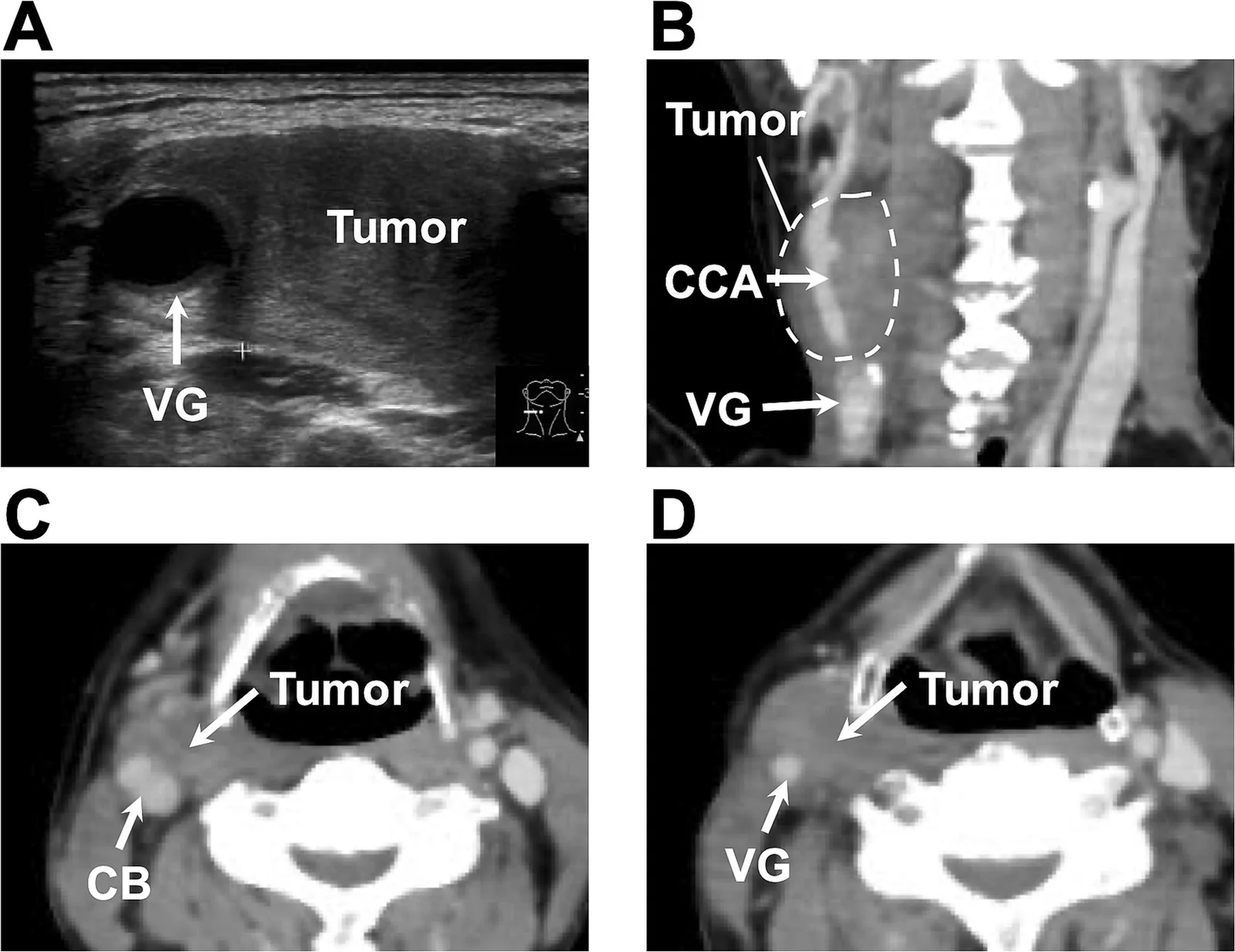
Clinical findings at the second recurrence. The tumor circumferentially surrounded the anastomosis between the common carotid artery and vascular graft. (A) Ultrasound examination. (B–D) Contrast-enhanced CT. CB, carotid bifurcation; CCA, common carotid artery; VG, vascular graft.

**Figure 4:**
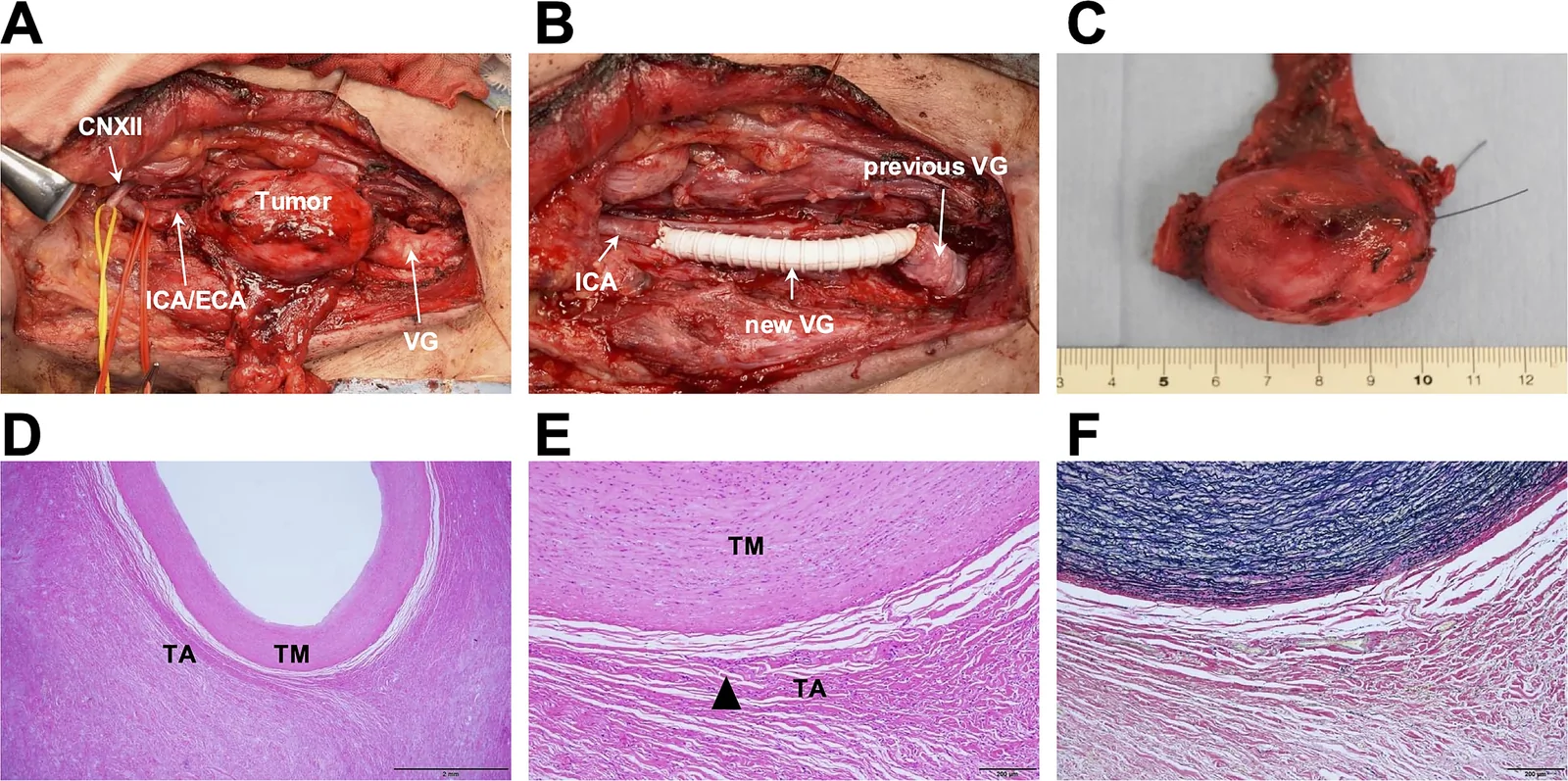
Surgical and histopathological findings at our second operation. (A) Before resection. The tumor surrounded the common carotid artery. (B) After vascular reconstruction. The internal carotid artery was connected to the previous vascular graft using a new vascular graft. (C) Tumor image. The tumor was resected en bloc. (D, E) Hematoxylin and eosin staining. The arrowhead represents tumor cells invading the adventitia of the common carotid artery. (F) Elastica van Gieson staining. CNXII, hypoglossal nerve; ECA, external carotid artery; ICA, internal carotid artery; VG, vascular graft; TA, tunica adventitia; TM, tunica media.

However, 2 years and 7 months after the surgery, the patient presented with a swelling on the right side of the neck. Ultrasound and contrast-enhanced CT showed the tumor circumferentially surrounding the anastomosis between the common carotid artery and vascular graft (Fig. 3). We planned repeat surgery to resect the recurrent tumor. We ligated the external carotid artery, performed en bloc tumor resection by cutting the distal vascular graft and proximal internal carotid artery. We reconstructed the segment between the internal carotid artery and the existing vascular graft using a heparin-bonded graft (Propaten, 6 mm, Gore), thereby completing repeat carotid artery reconstruction (Fig. 4A and B). Histopathological examination confirmed dedifferentiated liposarcoma with invasion of the carotid artery adventitia, while the surgical margin could not be assessed. After the reoperation, the patient had no new neurological symptoms and was discharged from the hospital on postoperative Day 9. He received adjuvant radiotherapy to the right neck (60 Gy in 24 fractions). He remains alive without evidence of recurrence 2 years and 3 months after repeat surgery.

## Discussion

Dedifferentiated liposarcomas are prone to recurrence, and complete resection remains the treatment of choice. Miura *et al*. [4] reported local recurrence in 3 of 4 cases of mediastinal dedifferentiated liposarcoma, all of which were successfully controlled by reoperation or radiotherapy. In our case, macroscopically complete resection of the tumor, including the involved adjacent organs, was achieved on both occasions, and the patient received adjuvant radiotherapy after both operations. The patient has remained free of recurrence for 2 years and 3 months since the final operation. Repeat resection with adjuvant radiotherapy may provide effective local control for recurrent dedifferentiated liposarcoma.

The overexpression of MDM2 and CDK4 is useful for distinguishing well-differentiated and dedifferentiated liposarcomas from other subtypes of liposarcomas [3]. Brahmi *et al*. [11] conducted the phase II trial to evaluate the safety/efficacy of ribociclib, a CDK4/6 inhibitor, administered in combination with siremadlin, an MDM2 inhibitor, in patients with advanced well-differentiated/dedifferentiated liposarcoma. They reported a 3-month progression-free survival rate of 41.2%, suggesting promising clinical activity of the regimen.

In patients with dedifferentiated liposarcomas invading the carotid artery, en bloc tumor resection and reconstruction of the carotid artery should be considered. There are few reports of dedifferentiated liposarcomas arising in the neck region. However, concerning head and neck cancers or carotid body tumors, these therapies contribute to prevention of neurological complications and better prognosis [12, 13]. Illuminati *et al*. [13] reported a retrospective study of vascular reconstruction for recurrent head and neck cancers showing carotid artery invasion. They reported that carotid artery resection with PTFE graft reconstruction provided effective local control without neurological complications and offered advantages over autografts in radiation tolerance and surgical invasiveness.

In our case, the recurrent tumor circumferentially surrounded the anastomosis between the artery and the vascular graft. As histopathology revealed tumor infiltration as far as the adventitia, we could not achieve complete resection of the tumor without vascular replacement. The patient developed neither postoperative neurological complications nor any graft-related complications after the adjuvant radiotherapy either on the first occasion or on the second. Even after vascular graft replacement and radiotherapy, repeat vascular graft replacement surgery may be considered where clinically indicated in patients presenting with recurrent dedifferentiated liposarcomas invading an artery.

## Conclusion

Repeat carotid artery reconstruction using a prosthetic vascular graft was successfully performed after recurrence following prior vascular graft replacement and postoperative radiotherapy, suggesting that this approach is feasible in selected patients.
